# Structure and Location Studies on Key Enzymes in Saponins Biosynthesis of *Panax notoginseng*

**DOI:** 10.3390/ijms20246121

**Published:** 2019-12-04

**Authors:** Pengguo Xia, Yujie Zheng, Zongsuo Liang

**Affiliations:** 1 Laboratory of Plant Secondary Metabolism and Regulation of Zhejiang Province, College of Life Sciences and Medicine, Zhejiang Sci-Tech University, Hangzhou 310018, China; zhengyj1025@163.com; 2 State Key Laboratory of Membrane Biology, Innovation Center for Structural Biology, School of Life Sciences, Tsinghua University, Beijing 100084, China

**Keywords:** *Panax notoginseng*, saponins biosynthesis, key enzymes, subcellular localization

## Abstract

*Panax notoginseng* is one of the most widely used traditional herbs for the treatment of various diseases, in which saponins were the main active components. At present, the research of *P. notoginseng* mainly focused on the discovery of new compounds and pharmacology. However, there were few studies on the molecular mechanism of the synthesis of secondary metabolites of *P. notoginseng*. In our study, four coding sequences (CDS) encoding the key enzymes involved in saponin biosynthesis were cloned, namely farnesyl diphosphate synthase (*FPS*), squalene synthase (*SS*), squalene epoxidase (*SE*), and dammarenediol-II synthase (*DS*), which contained open reading frame (ORF) of 1029 bp, 1248 bp, 1614 bp, and 2310 bp, and coded 342, 415, 537, and 769 amino acids, respectively. At the same time, their domains, secondary structures, three-dimensional structures, and phylogenetics trees were analyzed by kinds of bioinformatics tools. Their phylogenetics relationships were also analyzed. In addition, GFP (Green fluorescent protein) fusion genes were constructed by the plasmid transformation system to determine the subcellular localization. The results of subcellular localization showed that FPS, SE, and DS were mainly located in cytomembrane and its surrounding, while SS was located both in cytoplasm and cytomembrane. Our findings provided data demonstrating the expression patterns of genes involved in saponin biosynthesis and would facilitate efforts to further elucidate the biosynthesis of the bioactive components in *P. notoginseng*.

## 1. Introduction

*P. notoginseng* (Burk.) F.H.Chen, called ‘San-Qi’ in Chinese, is one of the most widely used traditional herbs for the treatment of various conditions, such as cardiovascular diseases, inflammation, various body pains, traumas, and internal and external bleeding due to injury [1]. Extensive chemical studies on this plant proved the dammarane-type saponins to be the main bioactive principles. By now, more than 80 saponins have been isolated from this root medicine, among which gisenosides Rg_1_, Re, Rb_1_, Rd, and notoginsenoside R_1_ are the five major components [2]. The ginsengsides Rg_1_, Rb_1_, and notoginsengside R_1_ are the main effective components responsible for blood circulation, nourishing blood, improving myocardial ischemia, anti-arrhythmia, antishock, sedative, increasing intelligence, anti-aging, anti-oxidation, and antitumor activities [3,4,5,6,7,8,9].

**Figure 1:**
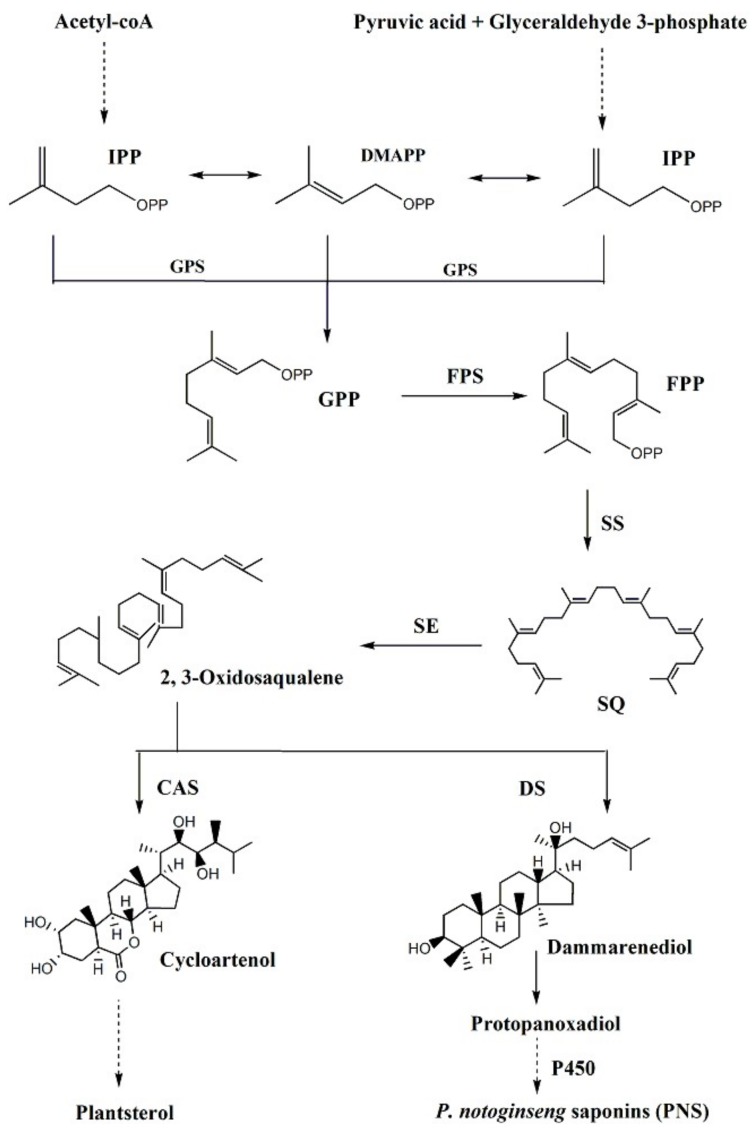
Biosynthetic pathways of saponins in *P. notogiseng*. The dotted lines indicate the multistep enzymatic reactions; GPS: Geranyl pyrophosphate synthase; GPP: Geranyl pyrophosphate; SQ: Squalene; CAS: Cycloartenol synthase.

Generally, the biosynthetic pathway of the plant dammarane-type saponins could be divided into three stages (Figure 1). The first stage leads to the synthesis of the isopentenyl pyrophosphate (IPP) and dimethylallyl diphosphate (DMAPP) through the 2-C-methyl-D-erythritol 4-phosphate (MEP) pathway and/or the mevalonate (MVA) pathway. In the second stage, IPP and DMAPP catalyze the synthesis of 2,3-oxidosqualene by isopentenyl transferase and anthracycline cyclase. The last stage involves a series of cyclization, oxidation, hydroxylation, and glycosylation reactions [10]. Enzymes involved in saponin biosynthesis have different subcellular localizations. All MEP pathway enzymes are located in plastids, whereas the MVA pathway enzymes can be in the cytosol or peroxisomes [11,12,13].

Farnesyl diphosphate synthase (FPS) catalyzes the sequential condensations of dimethylallyl diphosphate and geranyl diphosphate with isopentenyl diphosphate to produce FPP, thereby providing substrate for the synthesis of phytosterols, sesquiterpenes, and triterpenes [14]. Squalene synthase (SS) converts two FPP molecules into a C30 isoprenoid squalene, which is an essential substrate for the biosynthesis of cholesterol, steroid hormones, vitamin D, and triterpene [15]. The first oxygenation step in phytosterol and triterpenoid biosynthesis is performed by squalene epoxidase (SE), which catalyzes the epoxidation of the double bond of squalene to form 2, 3-oxidosqualene [16]. Dammarenediol-II synthase (DS) belongs to the oxidosqualene cyclase (OSC) family and catalyzes the cyclization of 2,3-oxidosqualene to form various secondary metabolites. Further diverse oxidation, hydroxylation, and glycosylation modifications are catalyzed by cytochrome P450-dependent monooxygenases (CYP450) and glycosyltransferases (GT), finally producing multiple ginsenosides with a variety of structures and biological activities.

Several genes associated with saponin biosynthesis had been cloned and characterized in many different species, and reports had indicated that changes in gene expression could enhance the production of secondary metabolites [17,18]. The research found that FPS was a rate-limiting enzyme in the saponin synthesis pathway, and overexpression of FPS in *P. notoginseng* cells can increase saponin content [19]. The overexpression of SS gene could significantly increase the content of phytosterols and triterpenoids in *Panax* species, which indicates that SS is a key enzyme in the metabolic flux of triterpene and sterol branches [20,21]. The transcriptome of the *P. notoginseng* root was analyzed and many genes involved in the triterpene saponin biosynthesis pathway were discovered, providing an essential foundation for further studies on *P. notoginseng* [22]. However, less comprehensive analysis is available regarding the expression patterns of the key genes for saponin biosynthesis in *P. notoginseng*.

This study was performed using two-year-old *P. notoginseng*. The full-length cDNA clones of *FPS*, *DS*, *SS*, and *SE* were obtained. To further confirm the functions of these genes, the characterized sequences of these predicted proteins in *P. notoginseng* were analyzed. Under the gene gun bombardment, the recombinant plasmids and the GFP alone were expressed in the onion epidermal cells, respectively. The subcellular localization of the four key enzymes in saponin synthesis was examined. Our findings provided data demonstrating the expression patterns of genes involved in saponin biosynthesis and would facilitate efforts to further elucidate the biosynthesis of the bioactive components in *P. notoginseng*.

## 2. Results

### 2.1. Isolation of Four Full-Length cDNA Sequences Involved in Saponin Biosynthesis

The extracted RNA was relatively complete. After being cloned into pMD19-T vector, a total of four independent clones of the PCR products were sequenced. Compared with NCBI, the genes we cloned were *FPS*, *SS*, *SE*, and *DS*, respectively. 

*FPS* contained a 1029 bp ORF encoding 342 amino acids (Figure S1A). The predicted FPS protein had an isoelectric point of 5.71, with the weight of 39.62 kDa. The SMART predicted that the protein has no specific domains such as repeats and transmembrane domains. *SS* contained a 1248 bp ORF encoding 415 amino acids (Figure S1B). The predicted SS protein had an isoelectric point of 6.64, with the weight of 47.17 kDa. The SS protein had two transmembrane domains at 281–303 aa and 385–407 aa, respectively. *SE* contained an ORF of 1614 bp in length and encoded 537 amino acids (Figure S1C). The protein had an isoelectric point of 8.56, with the weight of 59.18 kDa. The predicted SE protein possessed a low complexity region and four transmembrane domains at positions 20–42 aa, 69–91 aa, 467–489 aa, and 496–513 aa. *DS* contained a 2310 bp ORF encoding 769 amino acids (Figure S1D). The protein had an isoelectric point of 6.74, with the weight of 88.29 kDa. DS had only one transmembrane domain at 612–634 aa.

There was 14 rare codons in the ORF of *FPS*, and six single arginine rare codons; the ORF of *SS* had 32 rare codons and 11 single arginine rare codon; the ORF of *SE* had a total of 37 rare codons, and 19 single arginine rare codons, but there was a series of prosthetic and arginine, prosthetic tandem; *DS* had 52 rare codons, and 24 single arginine rare codons.

### 2.2. Prediction of Secondary and Tertiary Structures

**Figure 2:**
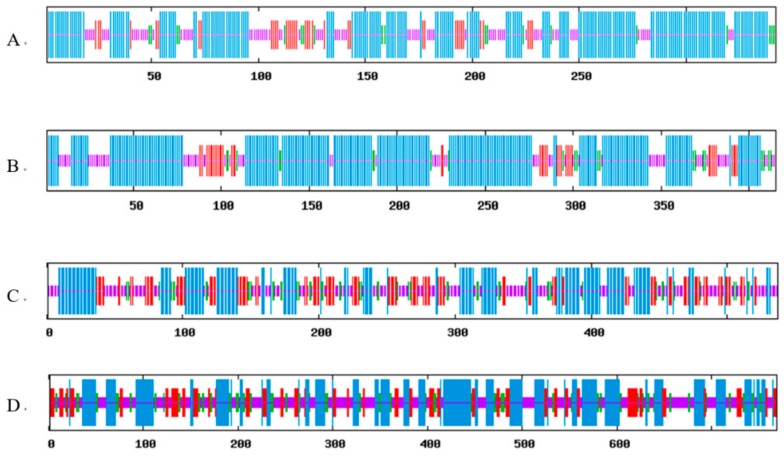
Secondary structure prediction of four key enzyme genes encoding protein in saponin synthesis: (**A**) farnesyl diphosphate (FPS); (**B**) synthase squalene synthase (SS); (**C**) squalene epoxidase (SE); (**D**) dammarenediol-II synthase (DS). Blue represents α-helix; green represents β-turn; purple represents random coil; and red represents extended strand.

The secondary structure is a conformational unit of protein structure, mainly including α-helix, β-sheet, β-turn, and random coil. The proteins encoded by *FPS*, *SS*, *SE*, and *DS* were predicted in secondary structures by SOPMA online tool (Figure 2). For *FPS*, the ratio of α-helix, β-turn, random coil, and extended strand were 58.19%, 5.56%, 25.44%, and 10.82%, respectively. The secondary structure of *SS* contained 65.30% of α-helix, 5.54% of β-turn, 20.00% of random coil, and 9.16% of extended strand. For *SE*, the ratio of α-helix, β-turn, random coil, and extended strand were 34.26%, 9.31%, 33.89%, and 22.53%, respectively. The secondary structure of *DS* contained 35.50% of α-helix, 11.57% of β-turn, 35.63% of random coil, and 17.30% of extended strand. 

**Figure 3:**
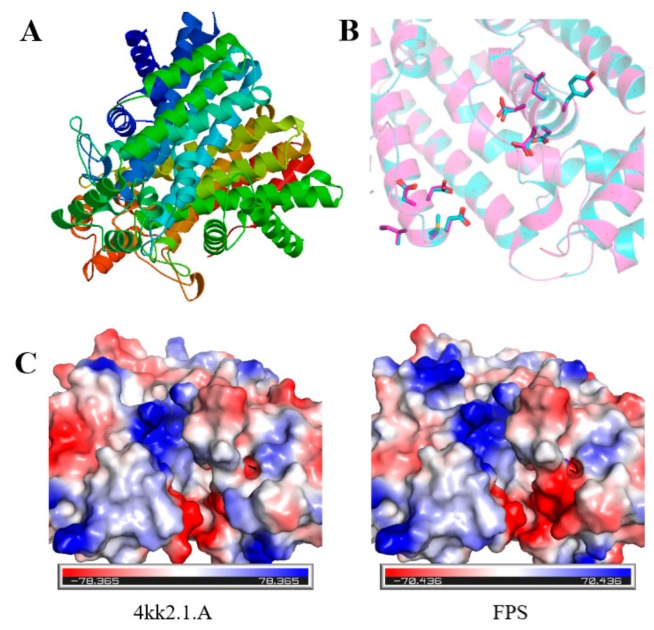
(**A**) 3-D structure prediction of FPS. (**B**) Superimposition of the template (cyan) and model (pink) structures. The catalytic residues are shown as stick. (**C**) The surface of the template (left) and model (right) depicted in vacuum electrostatics.

The 3-D structures of *FPS*, *SS*, *SE*, and *DS* were predicted by the SWISS-MODEL online tool, and molecular graphics figures were prepared with PyMOL. All prediction models were built based on ProMod Version 3.70 using X-ray at 2.20 Å. Among them, for the prediction of the three-dimensional structure of FPS (Figure 3A), the template number was 4kk2.1.A, the sequence identity was 84.71%, and the state of the oligonucleotide was homodimer. The sequence similarity to the template sequence was 56%, and the coverage was 99%. The predicted sequence was described as mononotepene synthase or farnesyl pyrophosphate synthase, which was consistent with the cloned gene. Since the catalytic residues of FPS (pink) were identical to that of template structure (cyan) (Figure 3B), the charge distributions as well as the size of active site were quite similar (Figure 3C).

**Figure 4:**
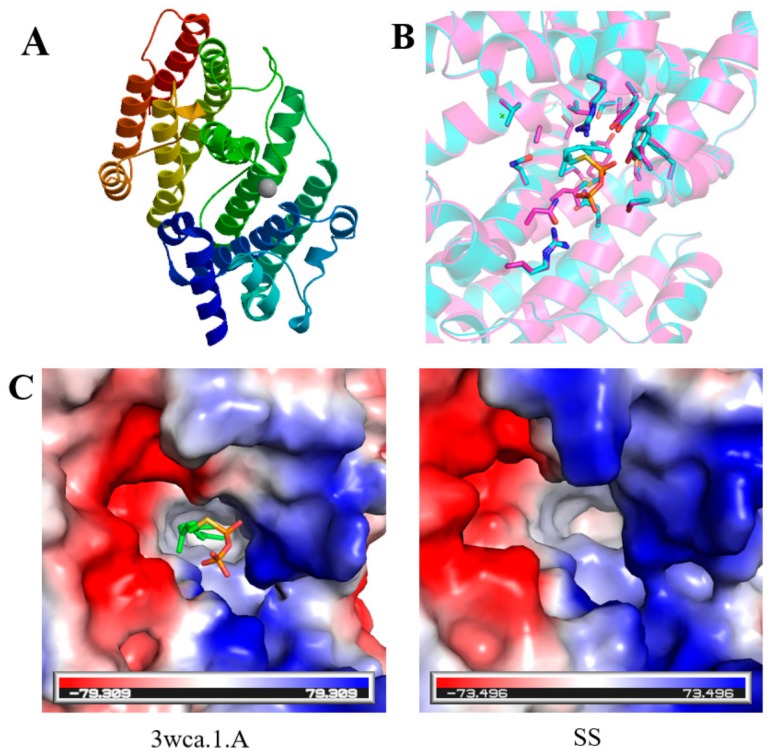
(**A**) 3-D structure prediction of SS. (**B**) superimposition of the template (cyan) and model (pink) structures. The catalytic residues are shown as stick. (**C**) the surface of the template (left) and model (right) depicted in vacuum electrostatics.

The prediction of the three-dimensional structure of SS was shown in Figure 4A. The template number of SS was 3wca.1.A, the sequence identity was 46.31%, the oligonucleotide was monomer, the similarity between the sequence and the template was 42%, and the coverage was 82%. The predicted sequence was described as farnesyl transferase. Since the catalytic residues of SS (pink) were highly conserved to that of template structure (cyan) (Figure 4B), the charge distributions as well as the size of active site were quite similar (Figure 4C).

**Figure 5:**
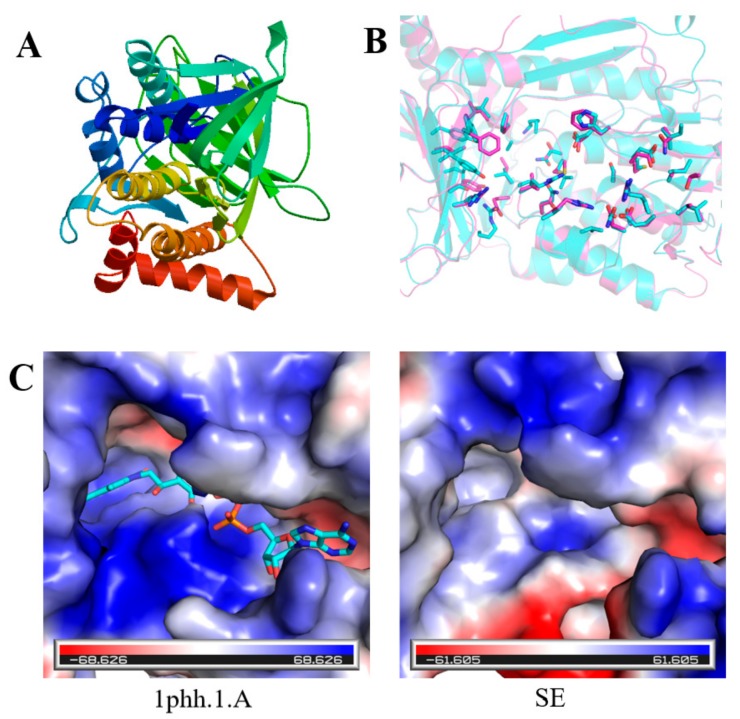
(**A**) 3-D structure prediction of SE. (**B**) superimposition of the template (cyan) and model (pink) structures. The catalytic residues are shown as stick. (**C**) The surface of the template (left) and model (right) depicted in vacuum electrostatics.

The prediction of the 3-D structure of SE is shown in Figure 5A. The template number of SE for the 3D-structure prediction was 1phh.1.A, the sequence identity was 19.74%, the oligonucleotide was homodimer, the sequence similarity to the template sequence was 28%, and the coverage was 71%. The predicted protein sequence was described as P-hydroxybenzoate hydroxylase. As shown in Figure 5B, the catalytic residues of SE (pink) and the template (cyan) were not conserved, thus the active site was quite different. The pocket of the template was more negative and deeper than that of SE (Figure 5C).

**Figure 6:**
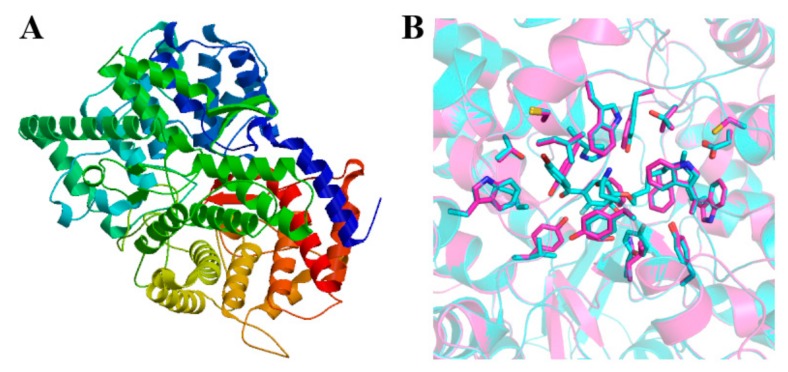
(**A**) 3-D structure prediction of DS. (**B**) superimposition of the template (cyan) and model (pink) structures. The catalytic residues are shown as stick.

For the prediction of the three-dimensional structure of DS (Figure 6A), the template number used was 1w6j.1.A, the sequence identity was 39.45%, the state of oligonucleotide was monomer, the sequence similarity was 40% with templates, and the coverage was 84%. The predicted protein was described as lanosterol synthase, which DS belonged to. As shown in Figure 6B, the residues in DS and the template were not conserved. The active site of the template was very small and not easy to display, so it was not drawn. DS did not have an active site corresponding to the template.

### 2.3. Sequence Homology and Phylogenetic Analysis

**Figure 7:**
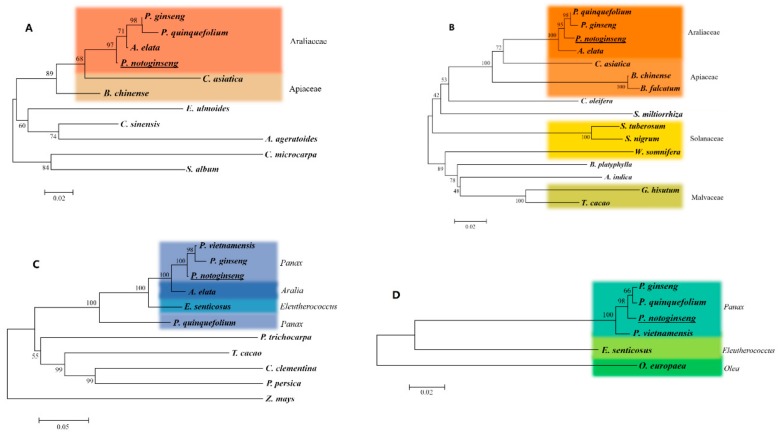
Phylogenetic tree analysis of gene sequence of saponins key enzymes in *P. notogiseng*: (**A**) *FPS*; (**B**) *SS*; (**C**) *SE*; (**D**) *DS*.

The predicted *FPS* protein exhibited 99%, 99%, and 96% identity with the *FPS* proteins of *Panax quinquefolium* (GQ401664), *Panax ginseng* (AAY87903), and *Bupleurum chinense* (EU400219), respectively. To determine the difference between the cloned nucleotide sequence and the same gene nucleotide in other plants, phylogenetic trees were constructed using NJ method. The species are showed in Table 1. According to the phylogenetic tree, *FPS* of *P. ginseng*, *P. quinquefolium*, *Aralia elata*, and *P. notoginseng*, all belonging to Araliaceae, formed a clade, which indicated that they had high homology (Figure 7A). 

The predicted *SS* protein exhibited 99%, 98%, and 96% identity with the *SS* proteins of *P. quinquefolium* (GU997681), *P. ginseng* (AB010148), and *A. elata* (GU354313), respectively. The phylogenetic tree found that *P. ginseng*, *P. quinquefolium*, *A. elata*, and *P. notoginseng* had high homology (Figure 7B). *SS* is encoded by a small gene family, and one, two, and three SS-encoding genes have been observed in *Medicago truncatula*, *Arabidopsis thaliana*, and *P. ginseng*, respectively.

The predicted *SE* protein showed high similarity with the corresponding enzymes from other plants, exhibiting 99%, 98%, and 96% identity to the *SE* proteins of *P. ginseng* (AB122078.1), *Panax vietnamensis* (KJ946469.1), and *A. elata* (GU354314.1), respectively. The phylogenetic tree showed that the *SE* of *P. ginseng*, *P. vietnamensis*, and *P. notoginseng*, which all belong to *Panax* formed a clade, while *P. quinquefolium* clustered outside of the clade (Figure 7C). 

The predicted *DS* protein exhibited 99%, 99%, and 97% identity with the *DS* proteins of *P. ginseng* (GU183405.1), *P. quinquefolium* (KC316048.1), and *P. vietnamensis* (KF306328.1), respectively. The phylogenetic analysis indicated that *DS* of *P. ginseng*, *P. vietnamensis*, *P. quinquefolium*, and *P. notoginseng* formed a clade (Figure 7D), which showed that they had high homology on saponin synthase. 

Ka/Ks is the ratio of nonsynonymous substitution (Ka) to synonymous substitution (Ks). It can be used to determine whether there is selective pressure on protein-coding genes, which is of great significance in evolutionary analysis. Ka/Ks results (Table S1) showed that the Ka/Ks > 1 only when *SS* of *P. quinquefolium* was compared with *P. ginseng*, indicating the presence of positive selection. When *FPS* of *A. elata* was compared with *P. ginseng* and *P. quinquefolium*, Ka/Ks > 0.5, which indicated that it was undergoing positive selection. It also maybe the reason that *P. quinquefolium* clustered outside of the clade in the phylogenetic tree. In addition, the *SE* of *P. quinquefolium* may had a large variation, resulting in it not being clustered with other *Panax* species. The analysis of phylogenetic tree found that plants of the same family and genus showed high similarity in key enzymes and final products of related metabolite synthesis, and related genes also showed high homology in nucleotide sequence.

### 2.4. Subcellular Localizations

**Figure 8:**
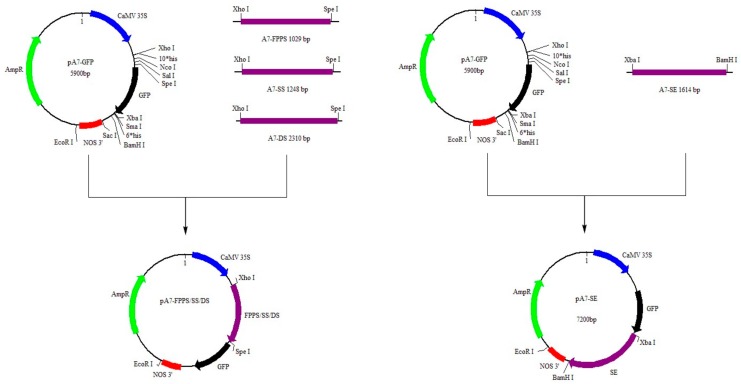
The build process diagram of the recombinant plasmid.

The results of the Nucleic acid quantitation instrument showed that the OD_260_/OD_280_ of all the extracted plasmids were between 1.7 and 1.9, indicating that the extracted plasmids met the requirements of the next experiment. The construction process of recombinant plasmid pA7-*FPS*/*SS*/*SE*/*DS* was shown in Figure 8. The results of PCR colony identification showed that the amplified bands of positive clones identified by *FPS*, *SS*, *SE*, and *DS* were about 1000 bp, 1200 bp, 1600 bp, and 2300 bp, respectively, which were consistent with the length of the previously cloned genes.

**Figure 9:**
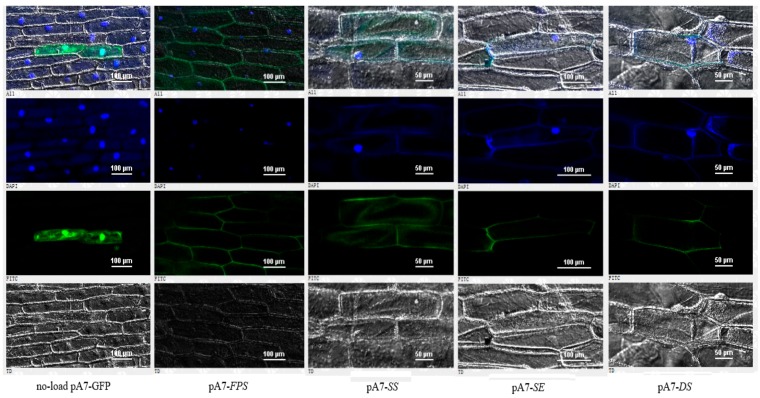
The subcellular localization results of FPS, SS, SE, and DS. Each image from the top to the next is the integration of image, blue fluorescence image, green fluorescence image, and transmission image. Each image from left to the right is no-load pA7-GFP, pA7-FPS, pA7-SS, pA7-SE, pA7-DS. Blue represents the fluorescence emitted by the nucleus, and green represents the result of GFP expression.

To examine the subcellular localization of four genes, the recombinant constructs and the GFP alone were introduced into onion epidermal cells by particle bombardment, respectively. As shown in Figure 9, SS were localized both in cytoplasm and cytomembrane, whereas GFP alone showed ubiquitous distribution in the whole cell. FPS, SE, and DS were mainly located in cytomembrane and its surrounding.

## 3. Discussion

The key enzymes in saponin synthesis are involved in the secondary metabolic process of *P. notoginseng* to affect the saponin synthesis regulation. At present, genes of key enzymes in the saponin synthesis pathway had been cloned from various medicinal plants containing saponin as a main component. Mevalonate-5- pyrophosphate decarboxylase, FPS, SS, SE, and DS had been cloned from *P. ginseng* [23,24]. The functions of these key enzymes were preliminarily verified by techniques such as overexpression, RNA silencing and RNA interference [25,26,27]. Key enzymes for plant secondary metabolic synthesis were also commonly found in other plants. The *FPS* had been cloned from *Gossypium arboretum*, *Cyclocarya paliurus*, *Siraitia grosvenorii*, and *Sonneratia alba* [28]. The *SS* had been cloned from *Trichosanthes rubriflos*, *Salvia miltiorrhiza*, *Dendrobium officinale*, and *Paris polyphylla* [29,30,31,32]. The *SE* had been cloned from *P. ginseng*, *P. quinquefolium*, and *E. senticosus* [33,34]. The analysis of phylogenetic tree found that plants of the same family and genus showed high similarity in key enzymes and final products of related metabolite synthesis, and related genes also showed high homology in nucleotide sequence.

Saponins are regarded as the main active ingredients of many medicinal plants, such as *P. ginseng*, *P. notoginseng*, *Polygala tenuifolia*, *Platycodon grandiflorus*, *Glycyrrhiza uralensis*, and *Anemarrhena asphodeloides*. The synthetic pathway of saponins also has some similarities in these medicinal plants. Upstream of a similar saponin synthesis pathway produced similar products and similar catalytically synthesized enzymes such as 3-hydroxy-3-methyl glutaryl coenzyme A reductase (HMGR), isopentenyl diphosphate isomerase (IPPI), and farnesyl diphosphate synthase. In this study, a green fluorescent fusion expression vector for the synthesis of several key enzymes in the saponins of *P. notoginseng* was constructed. The onion epidermal cells were transformed by gene gun. The results showed that the transformed fusion expression vector showed significant fluorescence in the onion epidermis, indicating that the system can be applied to other subcellular localization studies of saponins as the main secondary metabolite. In addition, the results of this study provided a basis and reference for *Panax* research.

## 4. Materials and Methods

### 4.1. Materials

Two-year-old *P. notoginseng* samples were collected from Kunming University of Science and Technology, which were identified by Prof. Xiu-ming Cui. All samples were thoroughly rinsed first with tap water and then with distilled water. After cleaning, a portion was taken for extraction of total RNA, and the rest was wrapped with tin foil paper, frozen in liquid nitrogen immediately, and stored at −80 °C until further processing.

### 4.2. RNA and DNA Isolation

Total RNA was isolated from sterile plants of *P. notoginseng* using the RNAprep pure Plant Kit (TIANGEN, Beijing, China). The RNA reversely transcribed according to the PrimeScript^TM^ RT reagent Kit (Takara, Shiga, Japan) to generate cDNA. Genomic DNA was isolated by using the Genomic DNA Isolation Kit (Cowin Biotech, Beijing, China).

### 4.3. Genes Cloning

The cDNA was used as a template, and the gene-cloning system was as follows: 2.0 μL of cDNA; 5 μL of 10×*Pfu* Buffer with MgSO_4_; 1 μL of 10 mmol/L dNTP; 0.6 μL of *Pfu* DNA polymerase; 37.4 μL of RNase free H_2_O; 2 μL of 10 μmol each of forward and reverse primer (Table 2) in a total volume of 50 μL. The PCR reaction was performed as follows: Preheating at 95 °C for 5 min, 38 cycles at 95 °C for 40 s, 40 s at annealing temperature and 72 °C for 2 min, then an extension at 72 °C for 10 min. The annealing temperatures of each gene were: FPS, 52 °C; SS, 55 °C; SE, 58 °C; DS, 55 °C. 

After the poly (A) tailing of PCR product, gene amplification was detected by 1% agarose-gel electrophoresis. After gel extraction of the gene, the product was ligated into pMD19-T vector and transformed into DH5α competent cells. Based on the antibiotic selection, the positive clones were sequenced by Sangon Biotech Company (Shanghai, China).

### 4.4. Bioinformatics Analysis

The chromas was used to view the sequencing results. DNAStar (7.1) and DNAMAN 9 were used to search the open reading frame (ORF) of four genes. According to the nucleotide sequence of each gene, the sequence of the corresponding amino acid was translated, and the isoelectric point of the amino acid, the distribution of the amino acid, and the size of the predicted protein were analyzed. SMART (http://smart.embl-heidelberg.de/) was used to analyze the domain of four genes. SOPMA (https://npsa-prabi.ibcp.fr/cgi-bin/npsa_automat.pl?page=/NPSA/npsa_sopma.html) was used to predict the secondary structure of the protein encoded by four genes. SWISS-MODEL program (http://swissmodel.expasy.org/) was used to create a 3-D structural model of the protein encoded by the four genes. Molecular graphics figures were prepared with PyMOL 2.3.0.

### 4.5. Phylogenetic and Genetic Evolutionary Analysis

In order to discuss the phylogenetic relationships of four genes, the sequence of *FPS*/*SS*/*DS*/*SE* were submitted to the NCBI database (https://blast.ncbi.nlm.nih.gov/Blast.cgi) to search the homologous sequences by BLAST. A total of 40 sequences were downloaded from the NCBI database. The NJ method constructed trees by clustering neighboring sequences in a stepwise manner. In each step of sequence clustering, it minimized the sum of branch lengths and thus examined multiple topologies. Using the NJ method, the phylogenetic tree was constructed by ClustalX 2.1 and MEGA4.0. The sequence used ClustalW 2 alignment and imported DNAsp5.10 to calculate Ka and Ks between sequences.

### 4.6. Construction of GFP Expression Vectors

The whole CDS of *FPS*/*SS*/*SE*/*DS* was amplified with forward and reverse primers using *Pfu* DNA Polymerase. The primers were shown in Table 3. The PCR reaction was performed as follows: Preheating at 95 °C for 5 min, 36 cycles at 95 °C for 40 s, 40 s at annealing temperature and 72 °C for extension time, then 72 °C for 15 min. The annealing temperatures of each gene were: FPS, 61 °C; SS, 60 °C; SE, 57 °C; DS, 58 °C. The extension time of each gene was: FPS, 2 min; SS, 3 min; SE, 3 min 30 s; DS, 5 min.

After the poly (A) tailing of PCR product, gene amplification was detected by 1% agarose-gel electrophoresis. After gel extraction of the gene, the product was ligated into pMD19-T Simple vector and transformed into DH5α competent cells. Based on the antibiotic selection, the positive clones were sequenced by Sangon Biotech Company (Shanghai, China).

For extraction of *FPS*/*SS*/*SE*/*DS* plasmid, 1 μL of the selected bacterial solution was added to 20 mL of LB medium containing antibiotic Amp, and then cultured at 37 °C for 12 to 16 h at 200 rpm shaker. For extraction of pA7-GFP plasmid, 1 μL of the bacterium containing the green fluorescent protein expression vector pA7-GFP was added to 20 mL of LB medium containing antibiotic Amp, and placed in a shaker at 200 °C for about 16 h at 37 °C. The plasmid was extracted by using the OMEGA Plasmid Kit (Shanghai, China).

The plasmid containing the four target genes and pA7-GFP vector were double-digested, respectively, and the reaction system was carried out by referring to the online DoubleDigest Calculator provided by Thermo Scientific (Shanghai, China). The reaction mixture of double digestion was shown in Table 4.

The purified 4 target gene fragments were ligated with the pA7-GFP vector plasmid fragment. The content ratio of the four target gene fragments to the pA7-GFP vector plasmid fragment in the reaction was 1:1 to 5:1. The reaction mixture of T4 DNA ligase was as follows: 1.0 μL of 10×T4 DNA ligase Buffer; 2.0 μL of pA7-GFP vector plasmid; 6.0 μL of purified 4 target gene fragments; 1.0 μL of T4 DNA ligase. The above reaction system was placed in a dry thermostat at 22 °C for 30 min, and then reacted at 16 °C overnight. The ligated product was transformed into DH5α competent cells. Based on the antibiotic selection, the positive clones were sequenced by Sangon Biotech Company (Shanghai, China).

The positive sequenced monoclonal colonies were added into 50 mL LB liquid medium containing Amp antibiotics, cultured at 37 °C for 14 h at 220 rpm, and the bacterial solution was used to extract recombinant plasmids.

### 4.7. Transformation of Onion Epidermal Cells by Gene Guns

Using the inner skin of the onion as the material, the inner bulb of the onion was cut with a blade and the inner epidermis (about 2×2 cm) was carefully peeled with tweezers and cultured in MS solid medium for 24 h. Gold powder and microparticle bombs were prepared, and the onion epidermal cells were bombarded with a gene gun under aseptic conditions; after bombardment, they were cultured in the dark at 25 °C for about 24 h.

The construct was confirmed by sequencing and used for transient transformation of onion epidermis via a gene gun. After 24 h of incubation, GFP fluorescence in transformed onion cells was observed under a confocal microscope (A1R, Nikon, Japan).

### 4.8. Nuclear Staining

The cultured material after transformation of the gene gun was stained with DAPI at 30 °C for 20 min, and then washed 3 times with PBS buffer. The expression of GFP was observed by laser scanning confocal microscope A1R (Nikon, Tokyo, Japan) at 488 nm.

## 5. Conclusions

In this study, the genes that are involved in saponin biosynthesis were cloned and detected, which provides a basic for the further discovery of downstream genes (such as CYP450 and GT) in *P. notoginseng*. The nucleotide sequence of FPS, SS, SE, and DS contained open reading frame (ORF) of 1029 bp, 1248 bp, 1614 bp, and 2310 bp, and coded 342, 415, 537, and 769 amino acids, respectively. According the SMART, there were two, four, and one transmembrane regions in the SS, SE, and DS, respectively. Furthermore, one low-complexity region was found in the SE. The analysis of the phylogenetic tree found that plants of the same family and genus showed high similarity in key enzymes and final products of related metabolite synthesis, and related genes also showed high homology in nucleotide sequence.

Under the gene gun bombardment, the recombinant plasmids and the GFP alone were expressed in the onion epidermal cells, respectively. The results showed that FPS, SE, and DS were mainly located in cytomembrane and its surrounding, while SS were located both in cytoplasm and cytomembrane. Our study will be useful to better understand the regulating mechanism of saponin production in *P. notoginseng* and provide a basic to improve contents of bioactive compounds in this traditional herbal.

## Figures and Tables

**Table 1:** Nucleotide sequences used for the phylogenetic tree analysis.

The Source Species	Accession No.	The Source Species	Accession No.
*P. ginseng*	AAY87903	*B. falcatum*	AY964186.1
*C. microcarpa*	AF390138	*S. nigrum*	JX984610.1
*C. asiatica*	AY787627	*G. hirsutum*	EF688567
*B. chinense*	EU400219	*S. miltiorrhiza*	JQ974834.1
*C. sinensis*	FS949364	*W. somnifera*	GU181386.1
*P. quinquefolium*	GQ401664	*P. ginseng*	AB122078.1
*A. elata*	HM219226	*P. vietnamensis*	KJ946469.1
*A. ageratoides*	JX424562	*E. senticosus*	JN228206.1
*E. ulmoides*	KC468536	*P. quinquefolium*	KC524469.1
*S. album*	KF011939	*A. elata*	GU354314.1
*P. ginseng*	AB010148	*T. cacao*	XM_007047548.1
*S. tuberosum*	JF802612.1	*C. clementina*	XM_006426079.1
*C. asiatica*	AY787628.1	*P. trichocarpa*	XM_006386402.1
*B. chinense*	GQ889266.1	*P. persica*	XM_007208396.1
*A. indica*	JQ327160.1	*Z. mays*	BT056068.1
*P. quinquefolium*	GU997681	*P. ginseng*	GU183405.1
*A. elata*	GU354313	*P. quinquefolium*	KC316048.1
*T. cacao*	XM_007041985	*P. vietnamensis*	KF306328.1
*B. platyphylla*	KP723830.1	*O. europaea*	AB291240.1
*C. oleifera*	JX914592.1	*E. senticosus*	JF818131.1

**Table 2:** Primers used in PCR amplification.

Primer	Primer Sequences (5′-3′)
FPSF	ATGAGCGATCTGAAGACGAGATT
FPSR	TTACTTTTGCCGCTTATATATCTTTCC
SSF	ATGGGAAGTTTGGGGGCAAT
SSR	TCACTGTTTGTTCGGTAGTAGGTTT
SEF	ATGAATTCATCTTCTTCTACTAGTAC
SER	TTAGTGAATGGGGGGAGCTCT
DSF	ATGTGGAAGCTGAAGGTTGCT
DSR	TTAAATTTTGAGCTGCTGGTGC

**Table 3:** Primer sequences used for construction of GFP expression vectors.

Primer	Primer Sequences (5′-3′)	Restriction Sites
A7-FPPSF	GGGGCTCGAGATGAGCGATCTGAAGA	*Xho*I
A7-FPPSR	GGGGGACTAGTGCCTTTTGCCGCTTATAT	*Bcu*I (*Spe*I)
A7-SSF	TTTTTTTTCTCGAGATGGGAAGTTTGGGGG	*Xho*I
A7-SSR	GGGACTAGTGCCTGTTTGTTCGGTAGT	*Bcu*I (*Spe*I)
A7-SEF	GGGGGGGTCTAGACATGAATTCATCTTCTT	*Xba*I
A7-SER	TTTGGATCCTTAGTGAATGGGGGGAGCT	*Bam*HI
A7-DSF	TTTTTTCTCGAGATGTGGAAGCTGAAGG	*Xho*I
A7-DSR	TTGGACTAGTGCAATTTTGAGCTGCTG	*Bcu*I (*Spe*I)

**Table 4:** Reaction mixture of double digestion.

Ingredient	Dosage (μL)	Ingredient	Dosage (μL)
10×Buffer Tango	2.5	10×Buffer Tango	2.5
*Xho*I	2.0	*Xba*I	1.0
*Bcu*I (*Spe*I)	0.5	*Bam*HI	1.0
*FPS/SS/DS*/pA7-GFP plasmid	≤1.0 μg	*SE*/pA7-GFP plasmid	≤1.0 μg
RNase Free H_2_O	up to 25	RNase Free H_2_O	up to 25

## Supplementary Materials

Supplementary materials can be found at https://www.mdpi.com/1422-0067/20/24/6121/s1.
