# Supplementary material for: Structure and Location Studies on Key Enzymes in Saponins Biosynthesis of Panax notoginseng
Source: Int J Mol Sci. 2019 Dec 4;20(24):6121. doi: 10.3390/ijms20246121 (PMC6940827; doi:10.3390/ijms20246121)
Supplement: Supplementary file 1 [file ijms-20-06121-s001.pdf]

International Journal of *Molecular Sciences*

MDPI

Supplementary Material

Supplementary Figure

Figure S1A

```
1     ATGAGCGATCTGAAGACGAGATTTCTGGAGGTGTACTCTGTTCTGAAATCCGAGCTACTC
1      M  S  D  L  K  T  R  F  L  E  V  Y  S  V  L  K  S  E  L  L
61    AACGACCCTGCTTTCGAGTTCACCGATGATTCTCGCCAATGGGTCGAGCGGATGCTGGAC
21     N  D  P  A  F  E  F  T  D  D  S  R  Q  W  V  E  R  M  L  D
121   TATAATGTGCCTGGAGGAAAGCTGAACCGAGGGCTGTCTGTTATTGACAGCTACAAGTTA
41     Y  N  V  P  G  G  K  L  N  R  G  L  S  V  I  D  S  Y  K  L
181   CTGAAAGAAGGAAAAGAACTAAGTGATGATGAAATTTTTCTTTCAAGTGCACTTGGTTGG
61     L  K  E  G  K  E  L  S  D  D  E  I  F  L  S  S  A  L  G  W
241   TGCATTGAATGGCTTCAAGCTTATTTTCTGGTGCTTGATGATATTATGGATAGCTCTCAT
81     C  I  E  W  L  Q  A  Y  F  L  V  L  D  D  I  M  D  S  S  H
301   ACGCGCAGAGGTCAACCCTGTTGGTTCAGATTACCTAAGGTTGGTATGATTGCCGTAAAT
101    T  R  R  G  Q  P  C  W  F  R  L  P  K  V  G  M  I  A  V  N
361   GATGGCATATTACTTCGCAACCATACCCCAAGGATTCTCAAGAAGCATTTCCGACAAAAG
121    D  G  I  L  L  R  N  H  T  P  R  I  L  K  K  H  F  R  Q  K
421   CCTTACTATGTGGATCTGTTGGATCTATTTAATGAGGTAGAATTCCAGACAGCTTGTGGA
141    P  Y  Y  V  D  L  L  D  L  F  N  E  V  E  F  Q  T  A  C  G
481   CAGATGGTAGATTTGATCACCACCCTTGTAGGAGAGAAAGACTTGTCGAAATACTCATTG
161    Q  M  V  D  L  I  T  T  L  V  G  E  K  D  L  S  K  Y  S  L
541   CCCATTCATCGCCGGATTGTGCAGTACAAAACTGCTTACTACTCATTTTACCTTCCAGTG
181    P  I  H  R  R  I  V  Q  Y  K  T  A  Y  Y  S  F  Y  L  P  V
601   GCCTGTGCACTTCTTATGTCAGGTGAAGATCTGGAGAAACATACTAATGTAAAGGACATA
201    A  C  A  L  L  M  S  G  E  D  L  E  K  H  T  N  V  K  D  I
661   CTTATTGAAATGGGAACCTATTTTCAAGTGCAGGATGATTATCTGGATTGCTTTGGTGCA
221    L  I  E  M  G  T  Y  F  Q  V  Q  D  D  Y  L  D  C  F  G  A
721   CCTGAGGTGATTGGGAAGATTGGCACAGATATTGAAGATTTCAAGTGCTCCTGGTTGGTA
241    P  E  V  I  G  K  I  G  T  D  I  E  D  F  K  C  S  W  L  V
781   GTAAAAGCACTGGAACTTTCTAACGAGGAACAAAAGAAGTTTTTACATGAGAACTATGGA
261    V  K  A  L  E  L  S  N  E  E  Q  K  K  F  L  H  E  N  Y  G
```

841 AAGGATGATCCGGCCTCTGTAGCAAAAGTGAAAGAGCTTTATAACACTCTCAAGCTTCAG

281 K D D P A S V A K V K E L Y N T L K L Q

901 GATGTATTTGCCGAGTATGAGAGCAAGAGCTATGACAAGTTGATCAAATTCATTGAAGCT

301 D V F A E Y E S K S Y D K L I K F I E A

961 CATCCGAGCCAAGCGGTACAAGCAGTGTTAAAATCATTCTTGGGAAAGATATATAAGCGG

321 H P S Q A V Q A V L K S F L G K I Y K R

1021 CAAAAGTAA

341 Q K *

Figure S1B

1 ATGGGAAGTTTGGGGGCAATTCTGAAGCATCCGGACGATTTCTATCCGTTATTGAAGCTT

1 M G S L G A I L K H P D D F Y P L L K L

61 AAATTTGCGGCTAGGCATGCGGAAAAGCAGATCCCTCCGGAGCCACACTGGGCCTTCTGT

21 K F A A R H A E K Q I P P E P H W A F C

121 TACTCTATGCTTCATAAGGTTTCTCGAAGTTTTGGCCTCGTCATTCAACAGCTCGGCCCT

41 Y S M L H K V S R S F G L V I Q Q L G P

181 CAGCTCCGCGATGCTGTATGCATTTTTTATTTGGTTCTTCGAGCACTTGACACTGTTGAG

61 Q L R D A V C I F Y L V L R A L D T V E

241 GATGACACAAGTATACCTACAGAGGTTAAAGTACCTATCTTGATGGCTTTTCATTGCCAC

81 D D T S I P T E V K V P I L M A F H C H

301 ATATATGATAAGGACTGGCACTTTTCATGTGGTACGAAGGAATACAAAGTTCTCATGGAC

101 I Y D K D W H F S C G T K E Y K V L M D

361 GAGTTTCATCATGTTTCTAATGCTTTTCTGGAGCTTGGAAACGGTTACCAGGAGGCAATA

121 E F H H V S N A F L E L G N G Y Q E A I

421 GAAGATATTACCATGAGAATGGGTGCAGGAATGGCAAAATTTATATGCAAGGAGGTGGAG

141 E D I T M R M G A G M A K F I C K E V E

481 ACAATAGATGATTATGATGAATATTGTCACTATGTAGCAGGACTTGTTGGATTAGGGTTG

161 T I D D Y D E Y C H Y V A G L V G L G L

541 TCAAAGCTCTTCCATGCCTCTGGGGCAGAAGATTTGGCTACAGATTTTCTGTCCAATTCA

181 S K L F H A S G A E D L A T D F L S N S

601 ATGGGTTTATTTCTCCAGAAGACAAACATAATTCGAGATTACTTGGAGGACATAAATGAG

201 M G L F L Q K T N I I R D Y L E D I N E

661 ATACCAAAGTCACGCATGTTTTGGCCTCGCCAGATTTGGAGTAAATATGTCGATAAACTT

221 I P K S R M F W P R Q I W S K Y V D K L

721 GAGGACTTAAAATATGAGGAAAACTCAGCCAAGGCAGTGCGGTGCCTAAATGACATGGTC

241 E D L K Y E E N S A K A V R C L N D M V

781 ACAAATGCTTTGGTTCATGCTGAAGATTGCCTAAAGTACATGTCTGACTTGCGAGATCCT

261 T N A L V H A E D C L K Y M S D L R D P

841 GCTATCTTCCGGTTCTGTGCAATACCACAGATTATGGCAATTGGAACACTAGCTTTATGC

281 A I F R F C A I P Q I M A I G T L A L C

901 TTCAACAACACTCAAGTCTTCAGAGGGGTAGTGAAAATGAGACGTGGTCTTACTGCTAAA

301 F N N T Q V F R G V V K M R R G L T A K

961 GTTATAGACCGAACAAAAACAATGTCAGATGTATATGGTGCTTTCTTCGATTTTTCTTGT

321 V I D R T K T M S D V Y G A F F D F S C

1021 TTGCTGAAGTCCAAGGTTGACAACAATGATCCCAATGCTACAAAAACTTTGAGCAGGCTA

341 L L K S K V D N N D P N A T K T L S R L

1081 GAAGCAATTCAGAAAACATGCAAGGAGTCTGGAACCCTGTCCAAAAGGAAATCATACATA

361 E A I Q K T C K E S G T L S K R K S Y I

1141 ATCGAGAGCGAGTCAGGACACAATTCAGCCCTGATTGCTATTATCTTCATTATACTAGCT

381 I E S E S G H N S A L I A I I F I I L A

1201 ATCCTTTATGCATATCTATCTTCAAACCTACTACCGAACAAACAGTGA

401 I L Y A Y L S S N L L P N K Q *

Figure S1C

1 ATGAATTCATCTTCTTCTACTAGTACTACTGATACGTTGCATTCTTTTATGGAAGCTCTG

1 M N S S S S T S T T D T L H S F M E A L

61 CTCATTGATCAATATTTTCTTGGATGGATCTTTGCTTTCCTTTTTGGGTTCCTGCTCTTG

21 L I D Q Y F L G W I F A F L F G F L L L

121 CTTAATTTCAAAAGGAAGAGAGAGAAAAATAATTCCACGGAGTTTGGAACAGATCATAGC

41 L N F K R K R E K N N S T E F G T D H S

181 AACGGATACTACATACCGGAAAATATTGCCGGAAGTACGGACGTCATCATCGTCGGCGCC

61 N G Y Y I P E N I A G S T D V I I V G A

241 GGGGTTGCTGGCTCTGCTCTTGCTTATACGCTTGCCAAAGATGGCCGGAGAGTTCATGTT

81 G V A G S A L A Y T L A K D G R R V H V

301 ATTGAGAGGGACTTAACTGAGCAAGACAGAATTGTAGGTGAACTTCTACAACCAGGAGGC

101 I E R D L T E Q D R I V G E L L Q P G G

361 TACTTGAAATTGATTGAATTAGGGCTAGAGGATTGTGTGAATGAAATCGATGCGCAACGA

121 Y L K L I E L G L E D C V N E I D A Q R

421 GTTTTTGGATATGCCCTTTACATGGATGGTAAAAACACCAGGCTTTCTTACCCCTTGGAG

141 V F G Y A L Y M D G K N T R L S Y P L E

481 AAATTTCATTCGGATGTAGCTGGAAGAAGCTTTCATAACGGCCGTTTTGTTCAACGAATG

161 K F H S D V A G R S F H N G R F V Q R M

541 AGGGAGAAAGCTGCATCACTTCCAAACGTAAGAATGGAACAGGGGACTGTTACATCTCTG

181 R E K A A S L P N V R M E Q G T V T S L

601 GTTGAGAAAAAGGGAAGTGTAAAAGGGGTGCAATACAAAACCAAGGATGGCCAAGAATTG

201 V E K K G S V K G V Q Y K T K D G Q E L

661 TCTGCATTTGCTCCTCTTACAATTGTTTGTGATGGTTGTTTTTCGAATCTCCGTCGCTCC

221 S A F A P L T I V C D G C F S N L R R S

721 CTCTGCAATCCCAAGGTGGAGGTGCCTTCGTGTTTTGTTGGTTTGATTTTGGAAAATATT

241 L C N P K V E V P S C F V G L I L E N I

781 GATCTTCCACATATAAACCATGGCCATGTCATTCTAGCAGATCCTTCTCCGATCTTGTTT

261 D L P H I N H G H V I L A D P S P I L F

841 TATAAAATTAGTAGTACCGAGATTCGCTGTTTGGTTGATTTGCCTGGACAAAAGGTGCCT

281 Y K I S S T E I R C L V D L P G Q K V P

901 TCTATTTCTAATGGGGAATTGGCTAATTATCTCAAGACAGTAGTAGCTCCTCAGGTTCCA

301 S I S N G E L A N Y L K T V V A P Q V P

961 AAACAGCTATATAACTCTTTCATAGCAGCAGTTGACAAAGGAAACATTCGAACCATGCCA

321 K Q L Y N S F I A A V D K G N I R T M P

1021 AACAGAAGCATGCCAGCTGATCCTCATCCAACTCCGGGTGCACTTCTATTAGGGGATGCT

341 N R S M P A D P H P T P G A L L L G D A

1081 TTCAATATGCGCCATCCTTTAACCGGCGGGGGAATGACAGTTGCTCTGTCTGATATTGTC

361 F N M R H P L T G G G M T V A L S D I V

1141 TTGATCCGGGATCTTCTTAGACCCTTATGCGATCTCCATGACTCATCAACCCTCTGCAAA

381 L I R D L L R P L C D L H D S S T L C K

1201 TATCTCGAATCCTTTTACACCCTTCGTAAGCCCGTGGCATCTACTATAAATACATTGGCA

401 Y L E S F Y T L R K P V A S T I N T L A

1261 GGTGCCCTTTATAAAGTTTTTTGTGCATCACCTGATAAAGCAAGGCAAGAAATGCGCAAT

421 G A L Y K V F C A S P D K A R Q E M R N

1321 GCGTGTTTTGATTATCTGAGCCTCGGAGGAATTTGTTCCCAAGGGCCAATTGCTTTACTT

441 A C F D Y L S L G G I C S Q G P I A L L

1381 TCTGGCCTAAACCCGCGTCCAATTAGCCTATTTCTCCACTTCTTTGCCGTGGCTATCTAT

461 S G L N P R P I S L F L H F F A V A I Y

1441 GGCGTTGGCCGCTTGTTGATTCCTTTTCCTTCACCAAAACGAATGTGGCTCGGCGCTAGA

481 G V G R L L I P F P S P K R M W L G A R

1501 TTGATTTTGGGTGCATCTGGAATTATTTTCCCTATTATAAAGTCAGAAGGACTTCGACAA

501 L I L G A S G I I F P I I K S E G L R Q

1561 ATGTTCTTCCCTGCAACTGTTCCTGCTTACTACAGAGCTCCCCCCATTCACTAA

521 M F F P A T V P A Y Y R A P P I H *

Figure S1D

1 ATGTGGAAGCTGAAGGTTGCTCAAGGAAATGATCCATATTTGTATAGCACTAACAACTTT

1 M W K L K V A Q G N D P Y L Y S T N N F

61 GTTGGCAGACAATATTGGGAGTTTCAGCCCGATGCTGGTACTCCAGAAGAGAGGGAAGAG

21 V G R Q Y W E F Q P D A G T P E E R E E

121 GTTGAAAATGCACGCAAGGATTATGTAAACAATAAAAAGCTACATGGAGTTCATCCATGC

41 V E N A R K D Y V N N K K L H G V H P C

181 AGTGATATGCTGATGCGCAGGCAGCTTATTAAAGAAAGTGGAATCGATCTCCTAAGCATA

61 S D M L M R R Q L I K E S G I D L L S I

241 CCGCCGGTGAGATTAGATGAAAACGAACAAGTGAACTACGATGCAGTTACAACCGCTGTG

81 P P V R L D E N E Q V N Y D A V T T A V

301 AAGAAAGCTCTTCGATTGAACCGGGCAATTCAAGCACACGATGGTCACTGGCCAGCTGAA

101 K K A L R L N R A I Q A H D G H W P A E

361 AATGCAGGCTCTTTACTTTATACACCTCCCCTTATCATTGCCCTATATATCAGCGGAACG

121 N A G S L L Y T P P L I I A L Y I S G T

421 ATTGACACTATTCTGACAAAACAACACAAGAAGGAACTGATTCGCTTCGTTTACAACCAT

141 I D T I L T K Q H K K E L I R F V Y N H

481 CAAAATGAGGATGGTGGATGGGGATCCTATATTGAGGGGCACAGCACGATGATTGGGTCA

161 Q N E D G G W G S Y I E G H S T M I G S

541 GTACTTAGCTTCGTGATGTTACGTTTGCTAGGAGAAGGATTAGCTGAATCTGATGATGGA

181 V L S F V M L R L L G E G L A E S D D G

601 AATGGTGCAGTTGAGAGAGGCCGGAAGTGGATACTTGATCATGGAGGTGCAGCCAGCATA

201 N G A V E R G R K W I L D H G G A A S I

661 CCCTCTTGGGGAAAGACTTATCTAGCGGTGCTTGGAGTATATGAGTGGGAAGGGTGCAAC

221 P S W G K T Y L A V L G V Y E W E G C N

721 CCGCTGCCCCCAGAATTCTGGCTTTTCCCTTCAAGTTTTCCTTTTCATCCAGCAAAAATG

241 P L P P E F W L F P S S F P F H P A K M

781 TGGATCTACTGCCGGTGTACCTACATGCCAATGTCGTATTTGTATGGGAAGAGATATCAT

261 W I Y C R C T Y M P M S Y L Y G K R Y H

841 GGACCAATAACCGATCTTGTTTTATCTTTGAGACAAGAAATTTACAACATTCCTTATGAG

281 G P I T D L V L S L R Q E I Y N I P Y E

901 CAGATAAAGTGGAATCAACAGCGCCATAACTGTTGCAAGGAGGATCTCTACTACCCTCAT

301 Q I K W N Q Q R H N C C K E D L Y Y P H

961 TCCCTTGTACAAGACCTGGTTTGGGATGGTCTTCACTACTTTAGTGAACCATTCCTCAAA

321 S L V Q D L V W D G L H Y F S E P F L K

1021 CGTTGGCCCTTCAACAAACTGCGAAAAAGAGGTCTAAAAAGAGTGGTTGAACTAATGCGC

341 R W P F N K L R K R G L K R V V E L M R

1081 TATGGTGCCACCGAGACCAGATTCATAACCACAGGAAATGGGGAAAAAGCTTTACAAATA

361 Y G A T E T R F I T T G N G E K A L Q I

1141 ATGAGTTGGTGGGCAGAAGATCCCAATGGTGATGAGTTTAAACATCACCTTGCTAGAATT

381 M S W W A E D P N G D E F K H H L A R I

1201 CCTGATTTCTTATGGATTGCTGAGGATGGAATGACAGTACAGAGTTTTGGTAGTCAACTA

401 P D F L W I A E D G M T V Q S F G S Q L

1261 TGGGACTGTATTCTTGCTACTCAAGCAATTATCGCCACCAATATGGTTGAAGAATACGGA

421 W D C I L A T Q A I I A T N M V E E Y G

1321 GATTCTCTTAAGAAGGCGCATTTCTTCATCAAAGAATCGCAGATAAAAGAAAATCCAAGA

441 D S L K K A H F F I K E S Q I K E N P R

1381 GGAGACTTCTTAAAAATGTGTCGACAGTTTACTAGAGGTGCGTGGACTTTCTCTGATCAA

461 G D F L K M C R Q F T R G A W T F S D Q

1441 GATCATGGTTGCGTTGTCTCGGACTGCACAGCTGAAGCACTAAAGTGCCTTCTGTTACTT

481 D H G C V V S D C T A E A L K C L L L L

1501 TCACAAATGCCACAGGACATTGCCGGAGAAAAACCTAAGGTTGAGCGATTATATGAGGCT

501 S Q M P Q D I A G E K P K V E R L Y E A

1561 GTGAATGTTCTTCTCTATTTGCAGAGTCGTGTAAGTGGTGGCTTCGCAGTTTGGGAGCCT

521 V N V L L Y L Q S R V S G G F A V W E P

1621 CCAGTTCCAAAACCATATTTGGAGATGTTGAATCCTTCAGAAATTTTTGCAGACATTGTT

541 P V P K P Y L E M L N P S E I F A D I V

1681 GTTGAGAGAGAGCACATTGAATGCACTGCATCTGTAATCAAAGGTCTGATGGCATTTAAA

561 V E R E H I E C T A S V I K G L M A F K

1741 TGCTTGCATCCTGGGCATCGTCAGAAAGAGATAGAGGATTCTGTGGCGAAAGCCATCCGT

581 C L H P G H R Q K E I E D S V A K A I R

1801 TATCTTGAAAGAAACCAAATGCCTGATGGTTCATGGTATGGCTTTTGGGGAATTTGTTTC

601 Y L E R N Q M P D G S W Y G F W G I C F

1861 CTCTATGGGACATTTTTTACCCTATCAGGGTTTGCTTCTGCTGGGAGGACTTATGACAAC

621 L Y G T F F T L S G F A S A G R T Y D N

1921 AGTGAAGCAGTTCGTAAGGGTGTTAAATTTTTCCTTTCAACACAAAATGAAGAAGGTGGT

641 S E A V R K G V K F F L S T Q N E E G G

1981 TGGGGGGAGAGTCTTGAATCATGCCCAAGCGAGAAATTTACACCACTCAAGGGAAACAGG

661 W G E S L E S C P S E K F T P L K G N R

2041 ACAAATCTAGTACAAACATCATGGGCTATGCTAGGTCTTATGTTTGGTGGACAGGCCGAG

681 T N L V Q T S W A M L G L M F G G Q A E

2101 AGAGATCCGACACCTCTGCATAGAGCAGCGAAGTTGTTGATCAATGCGCAAATGGATAAT

701 R D P T P L H R A A K L L I N A Q M D N

2161 GGAGATCTCCCTCAACAGGAAATTACTGGAGTATACTGTAAAAATAGTATGTTACATTAT

721 G D L P Q Q E I T G V Y C K N S M L H Y

2221 GCGGAGTACAGAAATATATTTCCTCTTTGGGCACTCGGAGAATATCGGAAACGTGTTTGG

741 A E Y R N I F P L W A L G E Y R K R V W

2281 TTGCCAAAGCACCAGCAGCTCAAAATTTAA

761 L P K H Q Q L K I *

Figure S1. The cDNA sequences and predicted amino acid sequences of four genes: (A)*FPS*; (B)*SS*; (C)*SE*; (D)*DS*. The sequences of shadow area represented the transmembrane regions.

Supplementary Table

Table S1. Ka/Ks analysis of four genes

Gene	Sequence 1	Sequence 2	Ka	Ks	Ka/Ks	Selective pressure
FPS	*A. elata*	*A. ageratoides*	0.0713	0.6121	0.116484	purify selection
		B. chinense	0.0189	0.4626	0.040856	purify selection
		C. sinensis	0.0608	0.4340	0.140092	purify selection
		C. asiatica	0.0157	0.3702	0.042410	purify selection
		C. microcarpa	0.0872	0.8200	0.106341	purify selection
		E. ulmoides	0.0633	0.7797	0.081185	purify selection
		P. quinquefolium	0.0062	0.0106	0.584906	purify selection
		S. album	0.0889	0.6938	0.128135	purify selection
		P. ginseng	0.0062	0.0106	0.584906	purify selection
		P. notoginseng	0.0031	0.0432	0.071759	purify selection

	A. ageratoides	*B. chinense*	0.0713	0.7699	0.092609	purify selection
		C. sinensis	0.0889	0.5501	0.161607	purify selection
		C. asiatica	0.0719	0.8531	0.084281	purify selection
		C. microcarpa	0.0850	1.0151	0.083736	purify selection
		E. ulmoides	0.0561	1.0203	0.054984	purify selection
		P. quinquefolium	0.0712	0.5928	0.120108	purify selection
		S. album	0.0949	0.9130	0.103943	purify selection
		P. ginseng	0.0712	0.5928	0.120108	purify selection
		P. notoginseng	0.0678	0.6395	0.106020	purify selection
	B. chinense	*C. sinensis*	0.0687	0.8108	0.084731	purify selection
		C. asiatica	0.0126	0.6106	0.020635	purify selection
		C. microcarpa	0.0751	0.9043	0.083048	purify selection
		E. ulmoides	0.0787	0.9641	0.081631	purify selection
		P. quinquefolium	0.0189	0.4461	0.042367	purify selection
		S. album	0.0942	1.0031	0.093909	purify selection
		P. ginseng	0.0189	0.4461	0.042367	purify selection
		P. notoginseng	0.0157	0.4648	0.033778	purify selection
	C. sinensis	*C. asiatica*	0.0530	0.6578	0.080572	purify selection
		C. microcarpa	0.0840	0.7411	0.113345	purify selection
		E. ulmoides	0.0464	0.6079	0.076328	purify selection
		P. quinquefolium	0.0607	0.4178	0.145285	purify selection
		S. album	0.0840	0.5663	0.148331	purify selection
		P. ginseng	0.0607	0.4178	0.145285	purify selection
		P. notoginseng	0.0574	0.4750	0.120842	purify selection
	C. asiatica	*C. microcarpa*	0.0769	0.8825	0.087139	purify selection
		E. ulmoides	0.0771	0.8709	0.088529	purify selection
		P. quinquefolium	0.0157	0.3553	0.044188	purify selection
		S. album	0.0960	0.9043	0.106159	purify selection

		P. ginseng	0.0157	0.3553	0.044188	purify selection
		P. notoginseng	0.0125	0.3549	0.035221	purify selection
	C. microcarpa	*E. ulmoides*	0.0752	1.4312	0.052543	purify selection
		P. quinquefolium	0.0880	0.7879	0.111689	purify selection
		S. album	0.0958	0.6989	0.137073	purify selection
		P. ginseng	0.0880	0.7879	0.111689	purify selection
		P. notoginseng	0.0837	0.7943	0.105376	purify selection
	E. ulmoides	*P. quinquefolium*	0.0632	0.7564	0.083554	purify selection
		S. album	0.0706	1.0295	0.068577	purify selection
		P. ginseng	0.0632	0.7564	0.083554	purify selection
		P. notoginseng	0.0599	0.8149	0.073506	purify selection
	P. quinquefolium	*S. album*	0.0897	0.6660	0.134685	purify selection
		P. notoginseng	0.0031	0.0323	0.095975	purify selection
	S. album	*P. ginseng*	0.0897	0.6660	0.134685	purify selection
		P. notoginseng	0.0853	0.6715	0.127029	purify selection
	P. ginseng	*P. notoginseng*	0.0031	0.0323	0.095975	purify selection
SS	*P. ginseng*	*S. tuberosum*	0.0896	0.8823	0.101553	purify selection
		C. asiatica	0.0387	0.3713	0.104228	purify selection
		B. chinense	0.0472	0.5214	0.090526	purify selection
		A. indica	0.0791	0.8650	0.091445	purify selection
		P. quinquefolium	**0.0047**	**0.0040**	**1.175000**	**positive selection**
		A. elata	0.0158	0.0434	0.364055	purify selection
		T. cacao	0.0821	0.7503	0.109423	purify selection
		B. platyphylla	0.0741	0.7662	0.096711	purify selection
		C. oleifera	0.0597	0.6221	0.095965	purify selection
		B. falcatum	0.0484	0.5707	0.084808	purify selection
		S. nigrum	0.0890	0.9407	0.094610	purify selection
		G. hirsutum	0.1009	0.9742	0.103572	purify selection

		S. miltiorrhiza	0.0835	0.9296	0.089824	purify selection
		W. somnifera	0.0947	0.9048	0.104664	purify selection
		P. notoginseng	0.0105	0.0288	0.364583	purify selection
	S. tuberosum	*C. asiatica*	0.0959	0.9989	0.096006	purify selection
		B. chinense	0.1125	1.0495	0.107194	purify selection
		A. indica	0.0939	1.0821	0.086776	purify selection
		P. quinquefolium	0.0844	0.8806	0.095844	purify selection
		A. elata	0.0884	0.8386	0.105414	purify selection
		T. cacao	0.0952	0.9689	0.098256	purify selection
		B. platyphylla	0.0928	0.9858	0.094137	purify selection
		C. oleifera	0.0789	0.9296	0.084875	purify selection
		B. falcatum	0.1166	1.0629	0.109700	purify selection
		S. nigrum	0.0165	0.1114	0.148115	purify selection
		G. hirsutum	0.1155	1.2450	0.092771	purify selection
		S. miltiorrhiza	0.1071	0.9940	0.107746	purify selection
		W. somnifera	0.0254	0.2196	0.115665	purify selection
		P. notoginseng	0.0860	0.8515	0.100998	purify selection
	C. asiatica	*B. chinense*	0.0590	0.6318	0.093384	purify selection
		A. indica	0.0690	1.0343	0.066712	purify selection
		P. quinquefolium	0.0338	0.3642	0.092806	purify selection
		A. elata	0.0381	0.3794	0.100422	purify selection
		T. cacao	0.0737	0.8637	0.085331	purify selection
		B. platyphylla	0.0741	0.9603	0.077163	purify selection
		C. oleifera	0.0609	0.7118	0.085558	purify selection
		B. falcatum	0.0602	0.6592	0.091323	purify selection
		S. nigrum	0.0973	1.0430	0.093289	purify selection
		G. hirsutum	0.0906	1.0194	0.088876	purify selection
		S. miltiorrhiza	0.0944	0.8994	0.104959	purify selection

		W. somnifera	0.1010	1.0260	0.098441	purify selection
		P. notoginseng	0.0375	0.3640	0.103022	purify selection
	B. chinense	*A. indica*	0.0926	0.9948	0.093084	purify selection
		P. quinquefolium	0.0423	0.5206	0.081252	purify selection
		A. elata	0.0454	0.5474	0.082938	purify selection
		T. cacao	0.0922	1.1278	0.081752	purify selection
		B. platyphylla	0.0936	1.0992	0.085153	purify selection
		C. oleifera	0.0863	0.7853	0.109894	purify selection
		B. falcatum	0.0035	0.0249	0.140562	purify selection
		S. nigrum	0.1119	1.1057	0.101203	purify selection
		G. hirsutum	0.1063	1.2541	0.084762	purify selection
		S. miltiorrhiza	0.0966	0.9990	0.096697	purify selection
		W. somnifera	0.1140	0.9496	0.120051	purify selection
		P. notoginseng	0.0423	0.5285	0.080038	purify selection
	A. indica	*P. quinquefolium*	0.0739	0.8634	0.085592	purify selection
		A. elata	0.0798	0.8529	0.093563	purify selection
		T. cacao	0.0759	0.5586	0.135875	purify selection
		B. platyphylla	0.0756	0.6409	0.117959	purify selection
		C. oleifera	0.0719	0.8260	0.087046	purify selection
		B. falcatum	0.0939	1.0073	0.093219	purify selection
		S. nigrum	0.0907	1.0382	0.087363	purify selection
		G. hirsutum	0.0804	0.8846	0.090889	purify selection
		S. miltiorrhiza	0.1051	1.2854	0.081764	purify selection
		W. somnifera	0.0922	1.0642	0.086638	purify selection
		P. notoginseng	0.0752	0.8628	0.087158	purify selection
	P. quinquefolium	*A. elata*	0.0111	0.0392	0.283163	purify selection
		T. cacao	0.0770	0.7490	0.102804	purify selection
		B. platyphylla	0.0691	0.7649	0.090339	purify selection

		C. oleifera	0.0547	0.6211	0.088070	purify selection
		B. falcatum	0.0435	0.5699	0.076329	purify selection
		S. nigrum	0.0838	0.9389	0.089253	purify selection
		G. hirsutum	0.0963	0.9649	0.099803	purify selection
		S. miltiorrhiza	0.0803	0.9347	0.085910	purify selection
		W. somnifera	0.0895	0.9030	0.099114	purify selection
		P. notoginseng	0.0058	0.0246	0.235772	purify selection
	A. elata	*T. cacao*	0.0796	0.7565	0.105221	purify selection
		B. platyphylla	0.0742	0.7393	0.100365	purify selection
		C. oleifera	0.0597	0.6004	0.099434	purify selection
		B. falcatum	0.0466	0.5983	0.077887	purify selection
		S. nigrum	0.0882	0.8891	0.099201	purify selection
		G. hirsutum	0.0951	1.0042	0.094702	purify selection
		S. miltiorrhiza	0.0871	0.9002	0.096756	purify selection
		W. somnifera	0.0935	0.8594	0.108797	purify selection
		P. notoginseng	0.0147	0.0477	0.308176	purify selection
	T. cacao	*B. platyphylla*	0.0667	0.5998	0.111204	purify selection
		C. oleifera	0.0678	0.7780	0.087147	purify selection
		B. falcatum	0.0935	1.2004	0.077891	purify selection
		S. nigrum	0.0985	0.9449	0.104244	purify selection
		G. hirsutum	0.0418	0.3770	0.110875	purify selection
		S. miltiorrhiza	0.1052	0.8511	0.123605	purify selection
		W. somnifera	0.1010	1.0022	0.100778	purify selection
		P. notoginseng	0.0789	0.7877	0.100165	purify selection
	B. platyphylla	*C. oleifera*	0.0690	0.7474	0.092320	purify selection
		B. falcatum	0.0975	1.1319	0.086138	purify selection
		S. nigrum	0.0935	1.0873	0.085993	purify selection
		G. hirsutum	0.0824	0.8398	0.098119	purify selection

		S. miltiorrhiza	0.1005	1.1374	0.088359	purify selection
		W. somnifera	0.0971	1.0555	0.091994	purify selection
		P. notoginseng	0.0691	0.7873	0.087768	purify selection
	C. oleifera	*B. falcatum*	0.0903	0.8435	0.107054	purify selection
		S. nigrum	0.0809	0.9771	0.082796	purify selection
		G. hirsutum	0.0836	1.1302	0.073969	purify selection
		S. miltiorrhiza	0.0871	0.8824	0.098708	purify selection
		W. somnifera	0.0904	0.9684	0.093350	purify selection
		P. notoginseng	0.0578	0.6254	0.092421	purify selection
	B. falcatum	*S. nigrum*	0.1160	1.1568	0.100277	purify selection
		G. hirsutum	0.1076	1.3651	0.078822	purify selection
		S. miltiorrhiza	0.1010	1.0547	0.095762	purify selection
		W. somnifera	0.1181	0.9328	0.126608	purify selection
		P. notoginseng	0.0435	0.5783	0.075220	purify selection
	S. nigrum	*G. hirsutum*	0.1175	1.0648	0.110349	purify selection
		S. miltiorrhiza	0.1091	0.9690	0.112590	purify selection
		W. somnifera	0.0242	0.2139	0.113137	purify selection
		P. notoginseng	0.0854	0.9074	0.094115	purify selection
	G. hirsutum	*S. miltiorrhiza*	0.1170	1.1067	0.105720	purify selection
		W. somnifera	0.1184	1.2992	0.091133	purify selection
		P. notoginseng	0.0963	1.0406	0.092543	purify selection
	S. miltiorrhiza	*W. somnifera*	0.1153	1.0328	0.111638	purify selection
		P. notoginseng	0.0829	0.9482	0.087429	purify selection
	W. somnifera	*P. notoginseng*	0.0911	0.8728	0.104377	purify selection
SE	*P. ginseng*	*P. notoginseng*	0.0048	0.0476	0.100840	purify selection
		P. vietnamensis	0.0048	0.0415	0.115663	purify selection
		E. senticosus	0.0166	0.2872	0.057799	purify selection
		P. quinquefolium	0.0405	0.5077	0.079772	purify selection

		A. elata	0.0136	0.0885	0.153672	purify selection
		T. cacao	0.1007	1.3729	0.073348	purify selection
		C. clementina	0.1096	1.4950	0.073311	purify selection
		P. trichocarpa	0.0987	1.7234	0.057271	purify selection
		P. persica	0.1014	1.6985	0.059700	purify selection
		Z. mays	0.1036	2.5305	0.040941	purify selection
	P. notoginseng	*P. vietnamensis*	0.0058	0.0416	0.139423	purify selection
		E. senticosus	0.0146	0.2709	0.053894	purify selection
		P. quinquefolium	0.0390	0.4833	0.080695	purify selection
		A. elata	0.0126	0.0789	0.159696	purify selection
		T. cacao	0.0995	1.4325	0.069459	purify selection
		C. clementina	0.1096	1.4584	0.075151	purify selection
		P. trichocarpa	0.0982	1.8056	0.054386	purify selection
		P. persica	0.1014	1.8259	0.055534	purify selection
		Z. mays	0.1042	2.4322	0.042842	purify selection
	P. vietnamensis	*E. senticosus*	0.0175	0.2876	0.060848	purify selection
		P. quinquefolium	0.0405	0.5143	0.078748	purify selection
		A. elata	0.0146	0.0918	0.159041	purify selection
		T. cacao	0.1017	1.4335	0.070945	purify selection
		C. clementina	0.1118	1.5222	0.073446	purify selection
		P. trichocarpa	0.1009	1.7916	0.056318	purify selection
		P. persica	0.1047	1.7646	0.059334	purify selection
		Z. mays	0.1058	2.2720	0.046567	purify selection
	E. senticosus	*P. quinquefolium*	0.0325	0.4621	0.070331	purify selection
		A. elata	0.0136	0.2184	0.062271	purify selection
		T. cacao	0.0919	1.4789	0.062141	purify selection
		C. clementina	0.1047	1.3429	0.077966	purify selection
		P. trichocarpa	0.0966	1.6281	0.059333	purify selection

		P. persica	0.0982	1.7108	0.057400	purify selection
		Z. mays	0.1021	2.3087	0.044224	purify selection
	P. quinquefolium	*A. elata*	0.0390	0.4495	0.086763	purify selection
		T. cacao	0.0999	1.2994	0.076882	purify selection
		C. clementina	0.1116	1.3829	0.080700	purify selection
		P. trichocarpa	0.1037	1.3576	0.076385	purify selection
		P. persica	0.1131	1.5832	0.071438	purify selection
		Z. mays	0.1036	2.3800	0.043529	purify selection
	A. elata	*T. cacao*	0.0980	1.3586	0.072133	purify selection
		C. clementina	0.1064	1.3379	0.079528	purify selection
		P. trichocarpa	0.0971	1.5967	0.060813	purify selection
		P. persica	0.1020	1.7609	0.057925	purify selection
		Z. mays	0.1020	2.1059	0.048435	purify selection
	T. cacao	*C. clementina*	0.0469	0.6161	0.076124	purify selection
		P. trichocarpa	0.0770	1.1582	0.066482	purify selection
		P. persica	0.0590	0.8074	0.073074	purify selection
		Z. mays	0.0834	2.1292	0.039170	purify selection
	C. clementina	*P. trichocarpa*	0.0845	1.2057	0.070084	purify selection
		P. persica	0.0634	0.8788	0.072144	purify selection
		Z. mays	0.0899	3.6556	0.024592	purify selection
	P. trichocarpa	*P. persica*	0.0871	1.0942	0.079602	purify selection
		Z. mays	0.0882	1.9038	0.046328	purify selection
	P. persica	*Z. mays*	0.0885	4.7311	0.018706	purify selection
DS	*P. notoginseng*	*P. ginseng*	0.0021	0.0383	0.054830	purify selection
		P. quinquefolium	0.0011	0.0266	0.041353	purify selection
		P. vietnamensis	0.0106	0.0500	0.212000	purify selection
		O. europaea	0.1655	1.1104	0.149045	purify selection
		E. senticosus	0.1386	0.6584	0.210510	purify selection

	P. ginseng	*P. quinquefolium*	0.0011	0.0189	0.058201	purify selection
		P. vietnamensis	0.0085	0.0499	0.170341	purify selection
		O. europaea	0.1627	1.1776	0.138162	purify selection
		E. senticosus	0.1376	0.6643	0.207135	purify selection
	P. quinquefolium	*P. vietnamensis*	0.0096	0.0620	0.154839	purify selection
		O. europaea	0.1640	1.1811	0.138854	purify selection
		E. senticosus	0.1388	0.6934	0.200173	purify selection
	P. vietnamensis	*O. europaea*	0.1585	1.1060	0.143309	purify selection
		E. senticosus	0.1375	0.6803	0.202117	purify selection
	O. europaea	*E. senticosus*	0.1287	1.2724	0.101147	purify selection
